# Prices of oncology medicines in European countries, Australia and New Zealand

**DOI:** 10.1186/2052-3211-8-S1-P16

**Published:** 2015-10-05

**Authors:** Sabine Vogler, Agnes Vitry, Zaheer Ud-Bin-Babar

**Affiliations:** 1 WHO Collaborating Centre for Pharmaceutical Pricing and Reimbursement Policies, Health Economics Department, Gesundheit Österreich GmbH (Austrian Public Health Institute), 1010 Vienna, Austria; 2 Quality Use of Medicines and Pharmacy Research Centre, Sansom Institute, School of Pharmacy and Medical Sciences, University of South Australia GPO, Adelaide, 5001, Australia; 3 Pharmacy Practice, School of Pharmacy Faculty of Medical and Health Sciences, University of Auckland, Private Mail Bag 92019, Auckland, New Zealand

## Background

Medicines are of major relevance for the treatment of cancer. They appear to be among high-cost medicines; however, their prices are not generally known. In this context, the study aims to survey the prices of oncology medicines in European countries, Australia and New Zealand and explore differences across the countries.

## Methods

Ex-factory prices per unit as for June 2013 of 31 oncology medicines in 16 European countries, Australia and New Zealand were surveyed and compared. Medicine price data for the 16 European countries were provided by the Pharma Price Information (PPI) service, and Australian and New Zealand medicine price data were retrieved from the respective Pharmaceutical Schedules. Official undiscounted list prices were taken into consideration. Price data refer mainly but not exclusively to the hospital sector and to medicines funded by the State.

## Results

Data availability was higher in the European countries compared with Australia and particularly New Zealand.

Oncology medicines are highly priced. None of the medicines surveyed had a unit price below EUR 10 in the 18 surveyed countries. Five medicines had an average unit ex-factory price between EUR 250 and EUR 1,000, and seven medicines had an average unit price above EUR 1,000.

The difference between the price of a medicine in the highest-priced country and the one in the lowest priced country varied between 28% and 233% except for one medicine with generics on the market (388%). A few medicines had lower outliers (particularly Greek and UK prices) and upper outliers (particularly prices in Switzerland, Germany and Sweden). Overall, Greek prices ranked at a low level, whereas Sweden, Switzerland and Germany showed price data in comparably high ranges.

No pattern was identified as to whether prices in Australia and New Zealand were high or low compared with European countries.

## Conclusions

While no relevant price differences of Australia and New Zealand in comparison with European countries were found, funding of oncology medicines appeared to be more restrictive in these two countries, and access to be granted at a later stage. However, these official list prices do not include discounts and similar arrangements that are in place for several of the surveyed medicines in a number of countries.

